# Antibacterial Activity of 7-Epiclusianone and Its Novel Copper Metal Complex on *Streptococcus* spp. Isolated from Bovine Mastitis and Their Cytotoxicity in MAC-T Cells

**DOI:** 10.3390/molecules22050823

**Published:** 2017-05-17

**Authors:** Mariana de Barros, Pedro Griffo Perciano, Marcelo Henrique dos Santos, Leandro Licursi De Oliveira, Éderson D’Martin Costa, Maria Aparecida Scatamburlo Moreira

**Affiliations:** 1 Department of Veterinary, Universidade Federal de Viçosa, Viçosa 36570-900, Brazil; mariana.barros@ufv.br; 2 Department of Chemistry, Universidade Federal de Viçosa, Viçosa 36570-900, Brazil; marcelo_hs@yahoo.com.br; 3 Department of General Biology, Universidade Federal de Viçosa, Viçosa 36570-900, Brazil; leandro.licursi@ufv.br; 4 Institute of Chemistry, Universidade Federal de Alfenas, Alfenas 37130-000, Brazil; edm_quimica@yahoo.com.br

**Keywords:** 7-epiclusianone, mastitis, bacterial activity, cytotoxicity, MAC-T, *Streptococcus* spp., *Rheedia brasiliensis*

## Abstract

Mastitis is an inflammation of mammary gland parenchyma that adversely affects bovine health and dairy production worldwide despite significant efforts to eradicate it. The aim of this work was to characterize the antimicrobial activity of 7-epiclusianone (7-epi), a compound extracted from the *Rheedia brasiliensis* fruit, its complex with copper against *Streptococcus* spp. isolated from bovine mastitis, and to assess their cytotoxicity to bovine mammary alveolar cells (MAC-T). The complex 7-epiclusianone-Cu (7-epi-Cu) was an amorphous green solid with optical activity. Its vibrational spectrum in the infrared region showed absorption bands in the high-frequency region, as well as bands that can be attributed to the unconjugated and conjugated stretching of the free ligand. The complex was anhydrous. One of the tested bacterial strains was not sensitive to the compounds, while the other three had MIC values of 7.8 µg mL^−1^ and minimum bactericidal concentration (MBC) values between 15.6 and 31.3 µg mL^−1^. These two compounds are bacteriostatic, did not cause damage to the cell wall and, at sub-inhibitory concentrations, did not induce bacterial adhesion. The compounds were not cytotoxic. Based on these results, 7-epi and 7-epi-Cu exhibited desirable antimicrobial properties and could potentially be used in bovine mastitis treatment.

## 1. Introduction

The human species has always used plants to control and eradicate diseases [1]. According to the World Health Organization, 70–80% of the world’s population depends exclusively on herbs for their primary health care [2]. Compounds isolated from plants are currently applied in modern therapies and play an important role in the synthesis of more complex molecules [3]. In this context, natural products have become an important source for the discovery of new therapeutic agents. Plants are especially rich in these types of compounds due to their wide chemical diversity [4] and represent an important source for new varieties of molecules [2].

**Figure 1:**
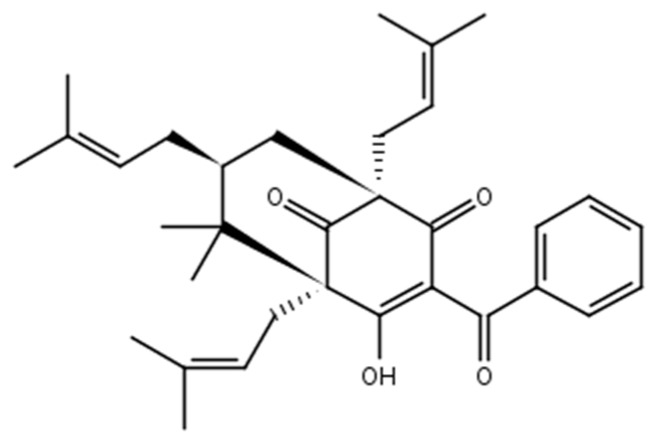
Chemical structure of 7-epiclusianone.

Plants from the genus *Rheedia* are often used in Brazilian folk medicine to treat disorders, such as constipation, rheumatism, inflammation, and pain [5]. *Rheedia brasiliensis* is native to the Amazonian region, planted all across Brazil, and popularly known as bacupari [6]. From its fruit, the molecule 7-epiclusianone (7-epi) was extracted (Figure 1) and shown to have a broad spectrum of biological activities [4] including toxicity to *Trypanosoma cruzi* trypomastigotes [7], vasodilation [8], anti-anaphylaxis [9], anti-HIV [10], antinociception, and anti-inflammation [11], among others. Vieira and colleagues [12] have investigated this compound using two epithelial cell lines: normal MDCK from canine kidneys and IIN-5 from a human carcinoma of the tongue. Their results clearly demonstrate that cellular reproduction time was reduced in the presence of 7-epi.

Compounds obtained from natural sources can be modified to make them more effective or less toxic [2]. A good strategy to increase antimicrobial activity is to complex active molecules with metals [13]. The antimicrobial properties of silver, copper, gold, titanium, and zinc all have distinct properties, potency, and activity that have been known and applied for centuries [14]. Traditional treatments utilize metallic ions with natural compounds. Historically, Chinese and Arabs used zinc to promote wound healing, while Egyptians used copper to sterilize water [15]. There is growing interest in Cu^2+^ complexes [16]. Among metallic ions, copper and its compounds have been used as effective agents against bacteria, fungi, viruses, and mollusks. Copper is a cheap metal and it is readily available, which makes it attractive for synthesis of new molecules [17]. There is a concern regarding the use of copper due to it toxicity, which occurs as an imbalance between influx and excretion resulting in liver cell damage [18]. Ruminants generally do not control copper uptake, storage, and biliary secretion, but cattle and goats can be considered copper-tolerant species [19].

Mastitis is an inflammation of mammary gland parenchyma that adversely affects bovine health and dairy production worldwide [20]. It is a complex disease with many factors [21]. This disease causes large financial losses in the milk production chain due to lesions on the secretory epithelial cells of mammary glands, which decrease milk production and excretion [22] The presence of pathogens in raw, unpasteurized milk increases the risk of their ingestion and transmission, as well as the potential ingestion of dangerous toxins [21,23]. There are literature reports of microorganisms that survive high pasteurization temperatures. [24]. Another public health concern is the extensive use of antimicrobials for mastitis treatment. This type of control has possible implications for human health by increasing the risk that resistant strains could enter the food chain or cause allergic reactions [21].

*Streptococcus* species, including *Streptococcus agalactiae* and *Streptococcus uberis*, are among the main mastitis pathogens [25]. Despite intense research and multiple approaches implemented over the past decades, bovine mastitis persists in milk herds [21]. Mastitis prevention and treatment comprise the majority of antimicrobial use in dairy cattle, resulting in substantial costs to the industry [26] and necessitating the search for new treatments. Additionally, unsuccessful treatment has prompted research into new approaches in order to reduce antimicrobial use [27]. 

The aim of this work was to characterize 7-epi and its metal complex 7-epiclusianone-copper (7-epi-Cu) and verify their antimicrobial activity on *Streptococcus* spp. isolated from bovine mastitis and assess their cytotoxicity in bovine mammary alveolar cells (MAC-T).

## 2. Results and Discussion

### 2.1. Chemistry

**Figure 2:**
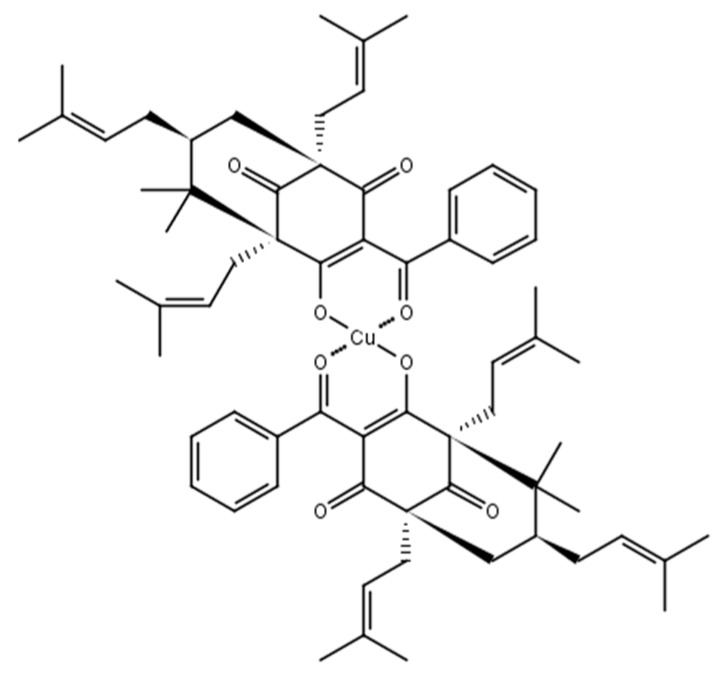
Chemical structure of the complex of 7-epiclusianone and Cu^2+^ (7-epi-Cu).

The compound 7-epi-Cu (Figure 2) was characterized by elemental analysis (CHN), atomic absorption, thermal analysis (TG), infrared (IR), nuclear magnetic resonance (NMR), ultraviolet/visible (UV-VIS) spectroscopy, and optical activity ([α]_D_).

The 7-epi-Cu complex was an amorphous green solid with an optical activity ${\left[\alpha \right]}_{D}^{25}$ = −70.1° (*c* 1.00, CHCl_3_). The results were consistent with a stoichiometry containing two 7-epi ligands for each metal center (1:2) (Cu^2+^: 7-epi) and C_66_H_82_CuO_8_: theoretical, C (74.30%), H (7.75%), Cu (5.96%); experimental, C (75.66%), H (7.60%), Cu (6.19%), which corresponded to a molar mass of 1065 Daltons.

**Figure 3:**
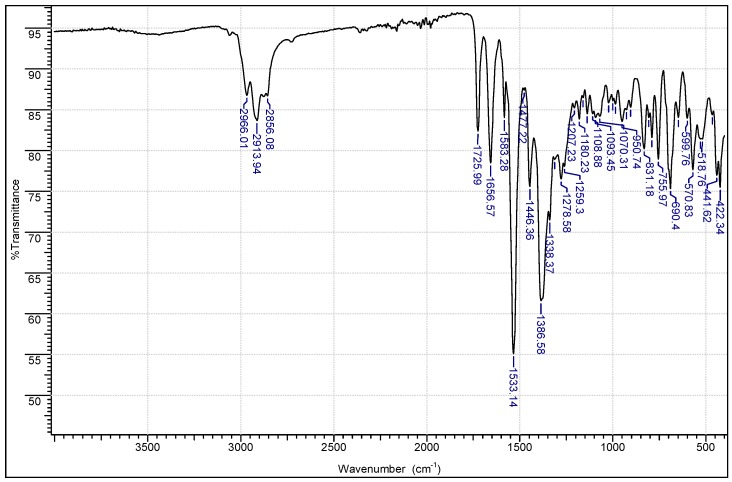
Infrared spectrum (IR) of 7-epiclusianone-copper (liquid film).

The vibrational IR spectrum (Figure 3) of 7-epi-Cu showed absorption bands in the high-frequency region of the spectrum at 2966 cm^−1^and 2913 cm^−1^, which we attributed to the aliphatic ν_(C-H)_ stretching of the methyl groups, as well as bands at 3062 cm^−1^ corresponding to aromatic ν_(C-H)_ from the binder. The absorption bands attributed to the unconjugated (1725 cm^−1^) and conjugated (1667 cm^−1^) stretching of the free ligand were displaced in the complex. The adsorption of the ν_(C=O)_ conjugate at 1667 cm^−1^ in the free ligand appeared to be displaced due to the small number of complexes (1656 cm^−1^) corresponding to the coordination of the oxygen atom to Cu^2+^. In addition, the 1583 cm^−1^ band that we attributed to the ν_(C=C)_ stretch of the aromatic ring of the 7-epi ligand was also observed in the complex with a band at 1533 cm^−1^. In fact, the band corresponding to the ν_(O-H)_ stretch of the free 7-epi ligand was not observed in the spectrum of the complex due to deprotonation of the ligand.

**Figure 4:**
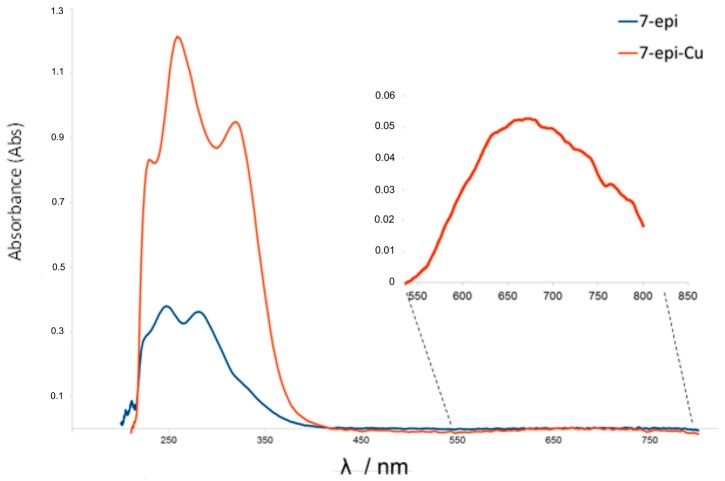
UV-VIS spectra of 7-epi (blue) and 7-epi-Cu (red) (0.04 mmol L^−1^ in methanol) showing the green color absorption of the 7-epi-Cu at 667 nm in the magnified region.

In the UV-VIS spectra (Figure 4) note a bathochromic shift in the complex (7-epi-Cu) when compared with the ligand (7-epi) in addition to a new band at 667 nm relative to the green color absorption showing that the complexation modified the chromosphere group in the ligand [6]. 

**Figure 5:**
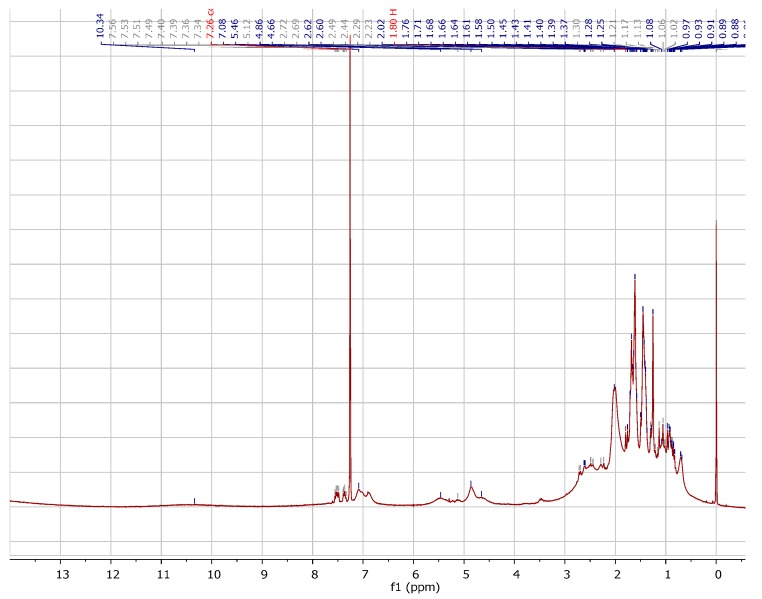
^1^H-NMR spectrum of 7-epi-Cu (300 MHz, CDCl_3_).

Comparative analyses of the ^1^H-NMR spectra (Figure 5) of 7-epi-Cu with 7-epi [6] were used to identify the signals corresponding to 8 CH_3_ (δ 0.6–1.6), 4-CH_2_ (δ 1.5–3.3), 1-CH (δ 1.0), 3 C=C-H (δ 4.5–5.5), 5 C=C-H_aromatic_ (δ 6.5–7.6) aromatic hydrogen and groups. The signals show a similar feature of paramagnetic spectra with distorted divide in the presence of Cu^2+^.

**Figure 6:**
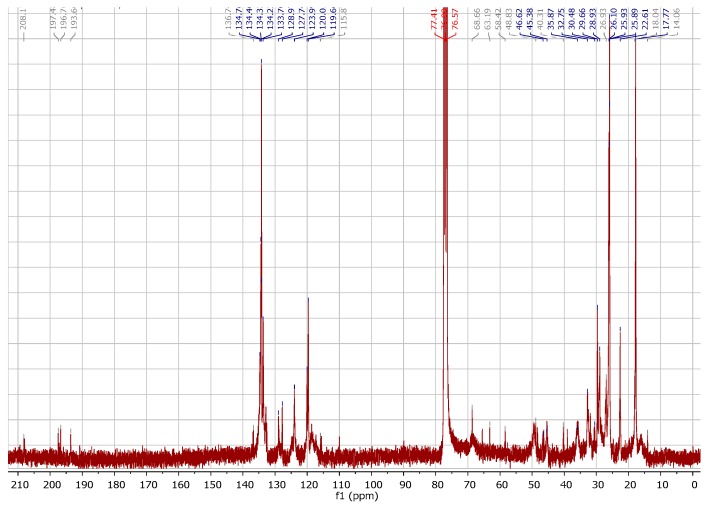
^13^C-NMR spectrum of 7-epi-Cu (75 MHz, CDCl_3_).

Comparative analyses of the ^13^C-NMR spectra (Figure 6) of 7-epi-Cu with 7-epi [6] were used to identify the signals corresponding to quaternary (8 sp^2^ and 2 sp^3^), methine (9 sp^2^ corresponding to 7 sp^2^ and 2 sp^3^), methylene (3 sp^3^), and methyl (six attached to 4 sp^2^ and two bonded to sp^3^) carbon atoms. Relative to carbonyl groups, were observed at δ 208.1 the non-conjugated carbonyl, and at δ 197.5, 196.7, and 193.6 the system conjugated carbonyl responsible for the complexation with the metal. The other signals were also similar to 7-epi [6].

**Figure 7:**
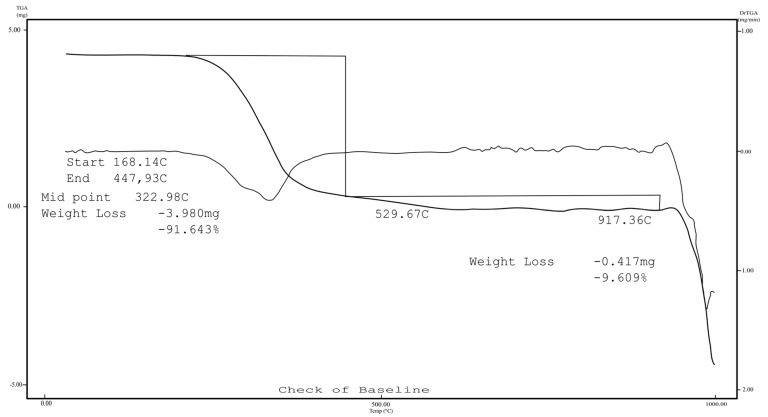
Thermal analysis (TG) curves of 7-epiclusianone-Cu.

TG was performed in a nitrogen atmosphere. The thermogravimetric curve shows that the complex was anhydrous, due to the existence of a plateau in stability up to 300 °C (Figure 7). Above this temperature, thermodecomposition of the complex began, with a visible loss in mass of 91.64% at 500 °C, which was consistent with the decomposition of the 7-epi ligands (calc. 93.92%). The observed 8.36% residue was tentatively attributed to CuO (7.45%).

### 2.2. Minimum Inhibitory Concentration (MIC) and Minimum Bactericidal Concentration (MBC)

Strains SA4038, SU959, and SU3580 had MICs of 7.8 μg mL^−1^ for both molecules. Strain SA3930 was resistant to them (Table 1).

According to Ríos e Recio [28], inhibitory activity values below 10 µg mL^−1^ for plant compounds are considered relevant. Cos et al. [29], in turn, described significant inhibitory activity as below 25 µg mL^−1^. Thus, based on these reports, 7-epi and 7-epi-Cu were significantly inhibitory (Table 1). The strain SA3930 was not used in the MBC assay since it showed no sensitivity to the tested compounds in the MIC test.

Metals have several mechanisms by which they induce microbial toxicity including protein dysfunction, production of reactive oxygen species and the depletion of antioxidants, disruption of membrane function, interference with nutrient assimilation, and genotoxicity [30]. Copper ions have been reported to be effective against bacteria and fungi isolated from mastitis [31], and its activity was expected to increase in a complex.

Comparing the activity of sulfathiazole and nimesulide complexed with or without copper, complexes with copper exhibited superior results and had activity, while copper alone was not active [15]. Santi et al. [13] evaluated the biological activity of free xylitol and xylitol complexed with copper or zinc and found that the MIC value for the complex was half that of free xylitol. In our study we found the same MIC value for 7-epi and its complex with copper, 7.8 µg mL^−1^.

Micro-organisms have genetic and biochemical mechanisms to combat metal toxicity, such as reduced absorption, efflux, extracellular sequestration, intracellular sequestration, repair, contouring pathways, and chemical modification [30]. Some of these mechanisms may explain the similarity in the antibacterial activity exhibited by benzophenone 7-epi and its complex with copper.

Another possibility for the similar activity values of 7-epi and 7-epi-Cu could be the existence of steric hindrance, which means that, in the spatial conformation of the molecule, the copper ion may not be accessible and is, therefore, unable to exert any antimicrobial activity. Furthermore, the size of the complex (1065 g mol^−1^) may prevent its action.

### 2.3. Time-Kill Curve

A time-kill curve provides concentration-time profiles that can be used in addition to in vivo studies to improve dosage planning [32].

**Figure 8:**
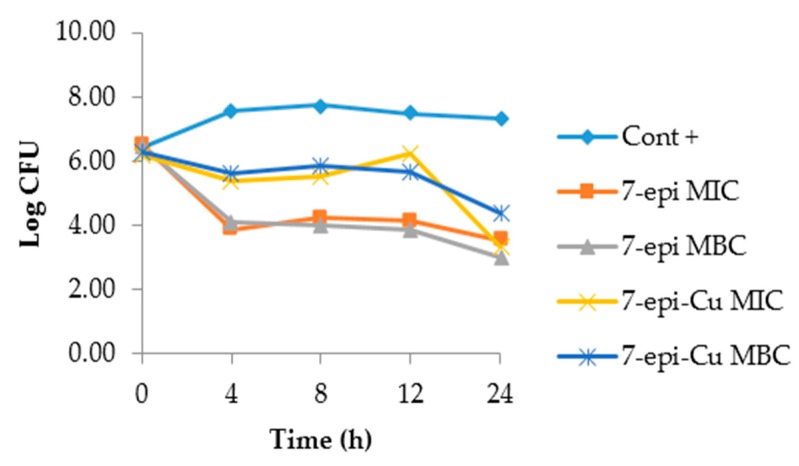
Time-kill curve of *Streptococcus agalactiae* isolated from mastitis after treatment with 7-epiclusianone (7-epi) and 7-epiclusianone-Cu (7-epi-Cu) at the minimal inhibitory concentrations (MIC = 7.8 μg mL^−1^) and minimal bactericidal (MBC = 15.6 and 31.3 μg mL^−1^ for 7-epi and 7-epi-Cu, respectively). Cont + = Growth curve for *S. agalactiae*.

**Figure 9:**
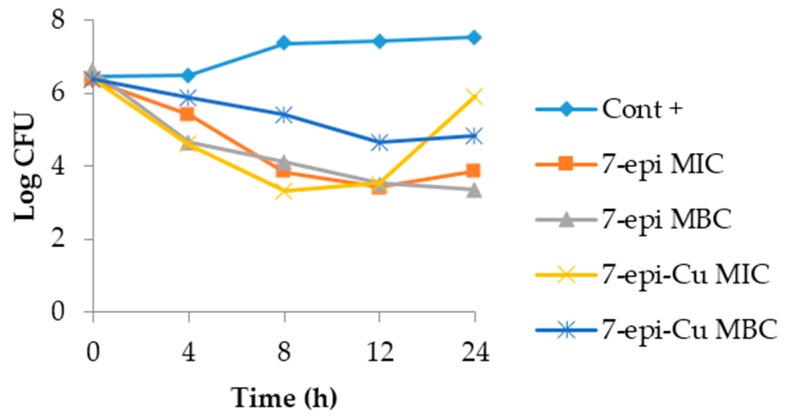
Time-kill curve of *Streptococcus uberis* isolated from mastitis after treatment with 7-epiclusianone (7-epi) and 7-epiclusianone-Cu (7-epi-Cu) at the minimal inhibitory concentrations (MIC = 7.8 μg mL^−1^) and minimal bactericidal (MBC = 31.3 μg mL^−1^). Cont + is the growth curve for *S. uberis.*

The dynamics of the time-kill curves for *S. agalactiae* and *S. uberis* treated with 7-epi and 7-epi-Cu revealed that these two compounds act by inhibiting proliferation of these bacteria and are, thus, bacteriostatic. This behavior was also observed in the CBM, which was a higher concentration than the MIC (Figure 8 and Figure 9).

The 7-epi compound and its complex with copper had the same pattern during the observation period where bacterial growth was inhibited. The behavior was similar for the two concentrations tested. This result demonstrates that the behavior of 7-epi and 7-epi-Cu are not time/dose-dependent. Pankey and Sabath [33] described reports of the most important clinical results for drugs with bactericidal activity relative to bacteriostatic agents and found that in vitro results are not sufficient to predict in vivo behavior. Furthermore, drug action is dependent upon the bacterial concentration and interaction with the immune system at the site of infection [34], thus, the bacteriostatic behavior of 7-epi and 7-epi-Cu against these two bacterial species is not a negative feature.

A theoretical complication of using bacteriostatic agents would be a relapse after infection treatment as the infectious agent is not totally eliminated. Nemeth et al. [34] looked for clinically-relevant distinctions between bactericidal and bacteriostatic antimicrobials and found no difference in the infection recurrence rate between these two categories.

Bactericidal compounds may lead to toxin release and the release of cell wall fragments, which leads to the increased release of prostaglandins and results in an exaggerated inflammatory response [33].

### 2.4. Protein Leakage Assay

The presence of protein in the supernatant is indicative of a disruption in the integrity of the bacterial cell wall [35]. There was no statistical difference in the amount of protein in the supernatant between treatments and controls at any of the studied time points (0, 3, 6, 12, 24, and 48 h) indicating there was no change in the cell wall. This result was expected as these compounds have a bacteriostatic mode of action and bacteriostatic antibiotics act by inhibiting bacterial protein synthesis [36].

### 2.5. Adherence Assessment

Subdoses (1/2 MIC) of 7-epi or 7-epi-Cu did not induce adhesion of *S. agalactiae* and *S. uberis* isolated from bovine mastitis.

The adhesion induction have clinical relevance because successful establishment of an infection by bacterial pathogens requires adherence to host cells and colonization of tissues [37]. Evidence suggests that antibiotics at sub-therapeutic doses stimulate bacteria to alter their metabolism and express virulence factors for adhesion and invasion [38,39,40]. These concentrations are present at the beginning and end of dosing regimens and during low dose treatments [41,42], as well as when antibiotics are used at concentrations below dose therapy (ppm) as additives in animal feed in order to promote growth and control diseases [43].

*Staphylococcus aureus* subjected to sub-doses of florfenicol and ciprofloxacin exhibited increased adherence to host cells. In *Escherichia coli* and *Pseudomonas aeruginosa*, aminoglycosides also increased adherence to host tissue [44].

Adhesion and internalization of *S. uberis* in mammary epithelial cells are important early events in the establishment of mastitis in cows [45]. Adhesion of *S. agalactiae* to host cells is a relevant stage of colonization [46,47].

Adhesion is the first step in the installation of an infection and also the first step in biofilm formation. Bacterial adhesion to the surface can be divided into different phases: primary and reversible adhesion, secondary and reversible adhesion, and biofilm formation [48]. A biofilm is defined as a cluster of cells enclosed in a self-produced matrix [49]. Due to the ability of biofilms to resist penetration by antimicrobials, biomedical research is focusing on the ability of bacteria to form biofilms. In clinical veterinary research, the characteristics of and therapeutic results in the treatment of mastitis suggest that biofilms are formed by mastitis-causing pathogens [50].

The compounds tested here did not induce adhesion. This result is clinically relevant.

### 2.6. Cytotoxic Effect

**Figure 10:**
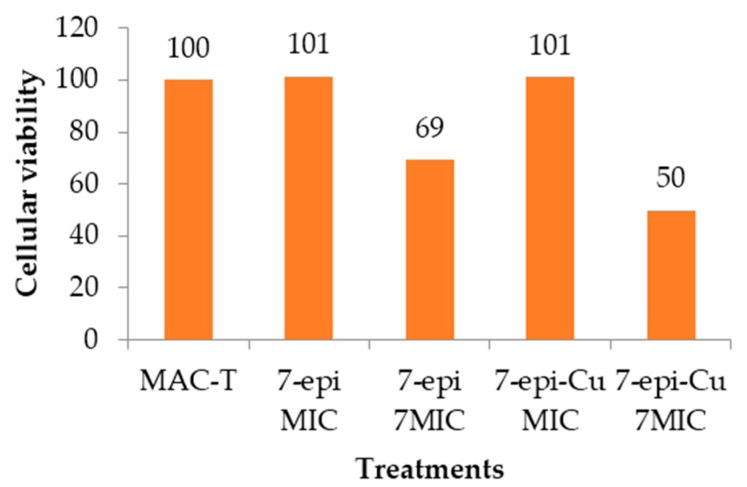
MAC-T viability by MTT assay. MAC-T cells were treated with 7-epiclusianone (7-epi) and 7-epiclusianone-copper (7-epi-Cu) for 24 h at the minimum inhibitory concentration (MIC) and seven times this concentration (7× MIC). The results were presented as cell viability in relation to the control (MAC-T), which was considered 100%.

A comparison of the values obtained from treated cells and controls in a MTT (3-(4,5-dimethylthiazol-2-yl)-2,5-diphenyltetrazolium bromide) test showed no statistical difference in the formation of formazan by mitochondrial dehydrogenases, indicating that the tested compounds were not cytotoxic. These values were similar even when using concentrations at 7× MIC (Figure 10).

Despite statistical results, microscopic analysis of cell cultures exposed to 7-epi and 7-epi-Cu showed that both compounds are not cytotoxic at their MIC in MAC-T cells. At this concentration there was no observed change in cell morphology. However, at 7× MIC the treated cells were more rounded than the control cells and had vacuolated cytoplasm. Moreover, in this treatment there was detachment and cell lysis. Therefore, further tests are needed to define the highest concentration at which the compounds are not toxic to this cell line.

## 3. Materials and Methods

### 3.1. Chemistry, Strains, and Culture Medium

The complex of 7-epi with Cu^2^+ [3-benzoyl-4-hydroxy-6,6-dimethyl−1,5,7-tris(e-methyl-2-butenyl)bicycle[3.3.1]non-3-ene-2,9-dione-copper], with the molecular formula C_66_H_82_CuO_8_, is a new compound obtained from 7-epi. The compound 7-epi was isolated and characterized by X-ray diffraction by Santos [6,51]. For the synthesis of 7-epi-Cu, 7-epi was weighed out, dissolved in ethanol, and added to potassium bicarbonate. The potassium bicarbonate ionized the benzophenone, which is a weak acid (pKa = 3.756) [52], and increased complexation. The metal ion Cu^2+^ was added to ionize the chelator molecule and there was a large excess of metal relative to benzophenone, which shifted the equilibrium toward complex formation. It was extremely important that there was no water in the solution because potassium bicarbonate reacts directly with water in a process called “hydrolysis” to produce hydroxyls that compete with the benzophenone for complexation with the metal. Subsequently the solution was filtered to remove excess potassium bicarbonate and chloroform was added. At that time, the ethanol was extracted with water in a funnel. At this stage, water did not affect the complexation because there was no excess of sodium bicarbonate to hydrolyze it, and also the whole complex was transferred to the chloroform phase. The chloroform phase was evaporated to obtain the complex. The compound 7-epi-Cu (Figure 2) was characterized by elemental analysis (CHN), atomic absorption, TG, IR, NMR, UV-VIS spectroscopy and [α]_D_.

At the time of utilization, compounds were solubilized in dimethyl sulfoxide (DMSO) and phosphate-buffered saline (PBS) pH 7.6 and heated in a water bath (Modelo 102, Fanem, São Paulo, SP, Brazil) at 60 °C (a temperature at which the compounds do not deteriorate). 

Two *Streptococcus agalactiae* strains, SA3930 and SA4038, and two *Streptococcus uberis* strains, SU959 and SU3580, at 10^5^ colony forming units (CFU) mL^−1^ in tryptic soy broth (TSB) were used. These strains belong to Empresa Brasileira de Pesquisa Agropecuária-Gado de Leite, and they were obtained from the milk of cows with mastitis in Zona da Mata Mineira, an important milk-producing region in Brazil.

#### 3.1.1. Instrumentation General Methods

UV-Visible spectra were determined on a spectrophotometer (UV−1601 PC, Himadzu, Tokyo, Japan). Infrared spectra were recorded on a Perkin Elmer Paragon 1000 FTIR spectrophotometer (Perkin Elmer, Waltham, MA, USA) (range from 400 to 4000 cm^−1^); ^1^H- and ^13^C-NMR spectra were run on a Varian Mercury (300 MHz) system (Varian, Palo Alto, CA, USA). The proton chemical shifts are reported relative to the signal of the residual chloroform in δ = 7.27 ppm and TMS (tetramethylsilane) at δ = 0.00 ppm. The ^13^C chemical shifts are reported using the signal in δ = 77 ppm from CDCl_3_ as a reference. Optical rotations were measured on a Perkin-Elmer-241 spectrophotometer (Perkin Elmer, Waltham, MA, USA); Elemental analysis: PE 2400 Series II CHNS/O Analyzer Perkin Elmer Instruments (Perkin Elmer, Waltham, MA, USA). The chromatography were performed by silica gel HPTLC (high-performance thin layer chromatography) plates (Sigma-Aldrich^®^, Newark, DE, USA), eluted with hexane:ethyl-acetate (70:30 *v*/*v*) to obtain the Rf (retention factor).

#### 3.1.2. 7-Epiclusianone-Cu Physical Data

Green amorphous powder (CHCl_3_); ${\left[\alpha \right]}_{D}^{25}$ −70.1° (*c* 1.0, CHCl_3_); *R*_f_ 0.30 (hexane:ethyl-acetate (85:15 *v*/*v*) in silica gel HPTLC); UV-Vis (MeOH, 0.4 mmol L^−1^) λ_max_ (Abs) 228 (0.82), 256 (1.20), 315 (0.84), 667 (0.06) nm; IR (film) ν_max_ 3062, 2966, 2913, 2856, 1725, 1656, 1533, 1387 cm^−1^; Elementary analysis: experimental, C (75.66%), H (7.60%), Cu (6.19%); calculated for C_66_H_82_CuO_8_, C (74.30%), H (7.75%), Cu (5.96%); ^1^H-NMR (300 MHz, CDCl_3_) δ (ppm): 0.6–1.6 (8 s, relative to 8 CH_3_ (δ),1.5–3.34 (m, relative to 4 CH_2_), 1.0 (m, relative to -CH), 4.5–5.5 (m, relative to 3 C=C-H) (δ),6.5–7.65 (m, relative to C=C-H_aromatic)_. ^13^C-NMR (75 MHz, CDCl_3_) δ (ppm): 208.1, 197.5, 196.7, 193.6, 136.7, 134.8, 134.3, 134.2, 133.7, 128.6, 127.7, 124.0, 120.0, 119.7, 115.87, 68.6, 63.1, 58.4, 48.8, 46.6, 40.3, 30.5, 29.6, 28.9, 26.9, 26.1, 25.9, 25.8, 22.6, 18.0, 17.7.

### 3.2. Minimum Inhibitory Concentration (MIC)

The four bacterial strains were submitted to minimum inhibitory concentration (MIC) tests by the microdilution broth as recommended by the *Clinical and Laboratory Standards Institute* [53]. Briefly, after compounds were solubilized two-fold serial dilutions were made in PBS (pH 7.6). Each well of a 96-well microtiter plate received 100 µL of bacterial culture and 100 µL of compound solution at the desired concentration (1–125 µg mL^−1^ for 7-epi and 0.5–62.5 µg mL^−1^ for 7-epi-Cu). A growth control was composed of 100 µL of bacterial culture with 100 µL of TSB, and the sterility control was 200 µL of TSB. Experiments were carried out in triplicate and repeated three times. A DMSO control was conducted previously and was shown to affect microbial growth at a concentration of 125 µg mL^−1^.

After inoculation, microtiter plates were incubated at 37 °C for 24 h before reading the results with a spectrophotometer (Titertek multiskan^®^ Plus-MKII, Flow Laboratories, Inc., McLean, VA, USA) at 550 nm. The MIC was designated as the lowest concentration capable of inhibiting bacterial growth, which was verified by comparing culture turbidity to the controls.

### 3.3. Minimum Bactericidal Concentration (MBC)

MBC was determined according to Dzotam [54]. After determining the MIC, 50 µL were taken from each well of the microtiter plate where no bacterial growth was observed and transferred to a new plate with 150 µL of TSB in each well. The plate was incubated at 37 °C for 24 h. After incubation, the plate was read with a spectrophotometer (Titertek multiskan^®^ Plus-MKII, Flow Laboratories, Inc., McLean, VA, USA) (550 nm).

MBC was designated as the lowest concentration where there was no bacterial growth, as verified by comparing the turbidity of the culture to controls. The experiment was performed in triplicate and repeated three times.

### 3.4. Time Kill Curve

Time kill curves for SA4038 and SU3580 were determined according to Li [55], with modifications. Cultures in broth were incubated with the two compounds at their MIC and MBC. At predetermined times (0, 4, 8, 12, and 24 h) a 50 µL aliquot was removed and diluted in ten-fold serial dilutions in 0.9% (*w*/*v*) autoclaved saline. Fifty microliters of each dilution was spread onto brain heart infusion agar in duplicate. A growth control was carried out in the absence of the test compounds. After incubating plates for 24 h at 37 °C, CFUs on plates with 25–300 colonies were counted. At the end, the number of counts were fit with a logarithmic function in Microsoft Excel^®^ 2010 software.

### 3.5. Protein Leakage Assay

Quantification of protein leakage from SA4038 and SU3580 exposed to 7-epi and 7-epi-Cu was determined according to Oyedemi et al. [56] and Bhande et al. [57], with modifications. The protein content in the supernatant of bacterial suspensions treated with the two compounds at their MIC collected at 0, 3, 6, 12, 24, and 48 h was determined by centrifugation at 3000× *g* for 30 min at 4 °C. The protein concentration was determined by the Bradford method [58] using bovine serum albumin to generate a standard curve. Controls were bacterial solutions in the absence of compound and compounds alone. Results were measured by spectrophotometry (Titertek multiskan^®^ Plus-MKII, Flow Laboratories, Inc., McLean, VA, USA) (550 nm).

### 3.6. Adherence Assessment

To evaluate if the tested compounds induced bacterial adhesion, we tested the sub-inhibitory concentration equal to ½ MIC [41]. Each well of a microtiter plate received 100 µL of bacterial culture and 100 µL of compound solution. The positive control consisted of 100 µL of culture with 100 µL of 0.9% (*w*/*v*) saline solution and the negative control was just TSB. Plates were incubated at 37 °C for 24 h, washed three times with distilled water and dried at room temperature. Subsequently, they were incubated with 250 µL of methanol for 15 min. After drying, wells were stained with a 2% crystal violet solution for 10 min, washed with autoclaved distilled water and dried at room temperature. Finally, 250 µL of 33% glacial acetic acid was added, and the results were read with a spectrophotometer (Titertek multiskan^®^ Plus-MKII, Flow Laboratories, Inc., McLean, VA, USA) at 550 nm.

The induction of adhesion was measured by absorbance at 550 nm, and the absorbance of the treated wells was compared to the controls. All procedures were performed in triplicate and repeated three times.

### 3.7. Cytotoxic Effect in MAC-T

The MAC-T cells were kindly provided by Yung-Fu Chang at the Medical College of Veterinary Medicine, Cornell University, Ithaca, NY. MAC-T cells were grown in flat-bottom 96-well culture plates in Dulbecco’s Modified Eagle Medium supplemented with 10% fetal bovine serum, penicillin (100 µg mL^−1^), and streptomycin (100 µg mL^−1^). Cells were incubated at 37 °C with 5% CO_2_ and 95% O_2_ for 24 h. Cell growth was visualized and monitored with an inverted microscope until they reached confluency (3 × 10^5^ cells) [41].

The compounds were used at concentrations equivalent to their MIC and 7× MIC. Cytotoxicity was assessed using an MTT assay as described by Al-Sheddi et al. [59]. Briefly, MAC-T cells were exposed to the compounds for 24 h. Then 10 µL of MTT (stock solution 5 mg mL^−1^ in PBS, pH 7.6) were added in 100 µL of media per well and incubated for 4 h. The supernatant was discarded and 200 µL of DMSO was added to each well to dissolve the crystals that had formed. The plate was gently stirred. The color that developed was read with a spectrophotometer (Titertek multiskan^®^ Plus-MKII, Flow Laboratories, Inc., McLean, VA, USA) at 550 nm. Cells not exposed to the compound served as a control. The experiment was carried out in quadruplicate.

### 3.8. Statistical Analysis

The averages obtained from the MTT test, protein leakage assay, and adherence assessment were compared by an analysis of variance (ANOVA), followed by Tukey’s method, using SASM-Agri software [60]. The significance level was 5% (*p* < 0.05).

## 4. Conclusions

The compounds 7-epi and 7-epi-Cu exhibited desirable antimicrobial characteristics and could potentially be used to treat bovine mastitis. Future work will focus on conducting clinical trials with these compounds. Although substantial differences were not observed between 7-epi and its new complex, 7-epi-Cu, the latter could still provide exciting results in other biological tests or with other microorganisms.

## Figures and Tables

**Table 1:** Minimum inhibitory concentration (MIC) and minimum bactericidal concentration (MBC) values for *Streptococcus agalactiae* (SA) and *Streptococcus uberis* (SU) isolated from bovine mastitis related by 7-epiclusianone (7-epi) and 7-epiclusianone-copper (7-epi-Cu).

Strains	MIC		MBC	
	Compounds (µg mL^−1^/µM)			
	7-epi	7-epi-Cu	7-epi	7-epi-Cu
SA3930	-	-	-	-
SA4038	7.8/15.5	7.8/7.3	15.6/31.1	31.3/29.3
SU959	7.8/15.5	7.8/7.3	31.3/62.2	31.3/29.3
SU3580	7.8/15.5	7.8/7.3	31.3/62.2	31.3/29.3
